# Resistance Exercise Counteracts Skeletal Muscle Atrophy in T2DM Mice by Upregulating FGF21 and Activating PI3K/Akt Pathway

**DOI:** 10.3390/biom16010003

**Published:** 2025-12-19

**Authors:** Xiaojie Ma, Zhijian Rao, Zhihai Jin, Yibing Lu, Zhitong Sun, Lifang Zheng

**Affiliations:** 1 College of Physical Education, Shanghai University, Shanghai 200444, China; maxiaojie@shu.edu.cn (X.M.); 1278493466@shu.edu.cn (Z.J.); luyibing0513@shu.edu.cn (Y.L.); 1287635496@shu.edu.cn (Z.S.); 2 College of Physical Education, Shanghai Normal University, Shanghai 200238, China; raoz19@shnu.edu.cn; 3 Exercise Biological Center, China Institute of Sport Science, Beijing 100061, China

**Keywords:** resistance exercise, skeletal muscle atrophy, fibroblast growth factor 21, type 2 diabetes mellitus, fibrosis

## Abstract

Decreased skeletal muscle mass and function are a serious complication of long-term diabetes, often leading to numerous adverse outcomes. The primary pathological features of diabetic sarcopenia include muscle fiber atrophy and interstitial fibrosis. Although resistance exercise (RE) has been reported to mitigate skeletal muscle atrophy in type 2 diabetes mellitus (T2DM), the underlying mechanisms remain unclear. Fibroblast growth factor 21 (FGF21), an exercise-induced cytokine, has been shown to protect against skeletal muscle atrophy at elevated levels. In this study, a T2DM mouse model was established through 12 weeks of high-fat diet feeding and intraperitoneal injection of streptozotocin (STZ) to investigate the effect and mechanism of RE on skeletal muscle atrophy in T2DM mice. Our results demonstrated that 8 weeks of RE significantly decreased body weight, fat mass, triglyceride (TG), low-density lipoprotein cholesterol (LDL-C), fasting blood glucose (FBG), and serum insulin levels in T2DM mice. RE also improved lean mass, glucose tolerance (IPGTT), and insulin tolerance (ITT). Additionally, RE increased skeletal muscle mass cross-sectional area (CSA) while attenuating fibrosis and inflammatory responses in skeletal muscle. Notably, RE upregulated FGF21 expression and activated the PI3K/Akt signaling pathway in diabetic skeletal muscle. RE promoted the phosphorylation of mTOR, 4EBP1, and p70S6K while suppressing the expression of the atrophy-related E3 ubiquitin ligases MuRF1 and MAFbx/Atrogin-1. Furthermore, RE inhibited lipid synthesis and enhanced both lipid oxidation and glucose utilization in skeletal muscle of T2DM mice. RE also improved mitochondrial biogenesis and dynamics in skeletal muscle of T2DM mice. In summary, 8 weeks of RE alleviated skeletal muscle atrophy in T2DM mice via activation of the FGF21/PI3K/Akt signaling pathway, which enhanced protein synthesis, improved glycolipid metabolism and mitochondrial quality control, and attenuated fibrosis and inflammation.

## 1. Introduction

Type 2 diabetes mellitus (T2DM) is a metabolic disease characterized by hyperglycemia and insulin resistance (IR), accounting for over 90% of all diabetes cases worldwide [1]. Skeletal muscle, which constitutes approximately 40% of adult body weight, plays a crucial role in maintaining glucose homeostasis and energy homeostasis [2]. Diabetes is a well-established risk factor for muscle atrophy [3], with diabetic individuals exhibiting a more pronounced decline in skeletal muscle mass and strength compared to non-diabetic counterparts. Notably, the prevalence of muscle atrophy is approximately 1.5 times higher in diabetic patients than in non-diabetics [4]. A hallmark of T2DM is IR, which is often accompanied by chronic inflammatory infiltration [5]. The interplay between IR and inflammatory cytokines disrupts normal muscle cell metabolism and function, leading to reduced protein synthesis and enhanced degradation. This metabolic dysregulation is further compounded by skeletal muscle fibrosis due to excessive extracellular matrix (ECM) deposition. These alterations contribute to the loss of skeletal muscle mass and impaired muscle fiber regeneration, ultimately resulting in muscle atrophy [6,7,8]. Skeletal muscle atrophy in T2DM increases the risk of weakness, falls, traumatic injuries, physical disability, and mortality [9]. Therefore, elucidating the underlying mechanism of diabetic muscle atrophy is essential for developing effective strategies to prevent its progression and improve clinical outcomes in T2DM patients.

Current guidelines recommend that patients with T2DM engage in resistance exercise (RE) at least twice weekly to achieve effective glycemic control. Previous studies have shown that RE alleviates skeletal muscle atrophy associated with heart failure [10] and age-related sarcopenia [11]. T2DM and obesity are also recognized as significant contributors to skeletal muscle atrophy. Studies indicate that RE can enhance skeletal muscle mass and increase muscle fiber cross-sectional area in T2DM rats by stimulating protein synthesis, thereby attenuating diabetes-induced muscle atrophy [12,13]. Nonetheless, the precise mechanism through which RE mitigates skeletal muscle atrophy in individuals with T2DM remains incompletely elucidated.

Fibroblast growth factor 21 (FGF21) is a metabolic regulator expressed in multiple tissues, including the liver, skeletal muscle, heart, and adipose tissue [14]. Studies have shown that the FGF21 expression is downregulated in the skeletal muscle of T2DM rats [15], and muscle-specific knockout of FGF21 in obese mice upregulated the expression of atrophy-related markers (MuRF1 and Atrogin-1) and inflammatory cytokines, exacerbating obesity-induced muscle atrophy [16,17]. These findings imply a protective role of FGF21 against skeletal muscle atrophy. The phosphatidylinositol 3-kinase (PI3K)/protein kinase B (Akt) signaling pathway plays central roles in regulating glycolipid metabolism [18], protein synthesis [19], mitochondrial function [20], inflammation [21], and fibrosis [22]. It is noteworthy that under IR/T2DM conditions, skeletal muscle exhibits not only chronic inflammatory infiltration, disordered glycolipid metabolism, and mitochondrial dysfunction [5,23], but also atrophic phenotypes, including accelerated protein degradation, increased interstitial fibrosis, and reduced fiber cross-sectional area (CSA) [24]. FGF21 functions as a pleiotropic factor that regulates glycolipid metabolism, mitochondrial homeostasis, and skeletal muscle integrity, while also suppressing inflammatory response and attenuating fibrotic progression [25,26]. Moreover, FGF21 has been shown to exert protective effects across multiple tissues and organ through regulation of the PI3K/Akt signaling pathway [27,28]. Although exogenous FGF21 supplementation has been shown to activate PI3K/Akt signaling and ameliorate metabolic disorders [27], it remains unclear whether RE influences endogenous FGF21 expression in skeletal muscle, thereby activating this pathway and attenuating muscle atrophy in T2DM. Elucidating this mechanism will provide novel insights into the molecular basis by which resistance exercise improves diabetic sarcopenia.

Based on the aforementioned evidence, we hypothesized that RE might upregulate endogenous FGF21 expression in the skeletal muscle of T2DM mice, which in turn activates the PI3K/Akt signaling pathway, thereby counteracting muscle atrophy by enhancing protein synthesis, improving glycolipid metabolism and mitochondrial function, and mitigating inflammation and fibrosis. To test this hypothesis, we established a T2DM mouse model and subjected the T2DM mice to an 8-week RE. Our study aimed to investigate the effects of RE on body composition, glycolipid metabolism, skeletal muscle mass, fibrosis, and inflammation. More importantly, we sought to determine whether the protective effects of RE against muscle atrophy were associated with the upregulation of FGF21 and the activation of the PI3K/Akt pathway. Our results demonstrated that RE effectively alleviated skeletal muscle atrophy in T2DM mice, and these benefits were concomitant with the upregulation of FGF21 and activation of the PI3K/Akt pathway, leading to enhanced anabolic signaling, improved mitochondrial quality control, and suppressed fibrotic and inflammatory responses. This study offers novel insights into the mechanisms by which resistance exercise counteracts diabetic sarcopenia.

## 2. Materials and Methods

### 2.1. Establishment of Mice Model of T2DM

A well-established mouse model of T2DM was induced by subjecting mice to 12 weeks of high-fat diet (HFD) feeding followed by a single streptozotocin (STZ) injection (100 mg/kg) [29,30] in order to mimic the natural progression of human T2DM, which is characterized by insulin resistance followed by progressive β-cell dysfunction. Six-week-old specific pathogen-free (SPF) male *C57BL/6* mice were obtained from Jicui Yaokang Biotechnology Co., Ltd. (Nanjing, China). The animals were housed under a 12 h light/dark cycle in a controlled environment with temperature maintained at 20–25 °C (daily fluctuation ≤ 3 °C) and relative humidity between 50% and 60%. Food and water were provided ad libitum. After one week of acclimatization, mice were randomly assigned to two groups: a control group (CON, *n* = 11) fed a standard diet (3.6 kcal/g, 4.8% kcal from fat), and a high-fat diet group receiving a high-fat diet (5.24 kcal/g, 60% kcal from fat; supplied by SYSE Ltd., Changzhou, China). Following 12 weeks of dietary intervention, T2DM was induced by intraperitoneal injection of streptozotocin (STZ, Sigma-Aldrich, St. Louis, Missouri, USA) at a dose of 100 mg/kg. One-week post-injection, fasting blood glucose (FBG) was measured using a Roche glucose meter (Berlin, Germany); the first drop of blood was discarded. Mice with FBG ≥ 13.8mmol/L were considered successful T2DM models. These diabetic mice were then randomly divided into a sedentary group (SED, *n* = 11) and a resistance exercise group (RE, *n* = 11). All animal experimental techniques have been supported by the Ethics Committee of Shanghai University (Approval Number: 2025-005).

### 2.2. Exercise Protocol

The RE protocol was designed based on well-established models known to induce muscle hypertrophy and metabolic improvements in rodents [31,32]. The training was conducted using a 1 m-high ladder inclined at 85° with 1 cm grid. Mice in the RE group first underwent a 7-day adaptation period of unloaded climbing once daily. The formal 8-week training regimen, performed three times per week, consisted of 3 sets of 5 repetitions per session, with 60 s rest intervals between sets. A progressive overload was applied by tail weighting as follows: 30% of body weight (BW) in week 1, 55% BW in week 2, 80% BW in weeks 3–4, 90% BW in weeks 5–6, and 100% BW in weeks 7–8. This specific duration and frequency were selected based on our previous study, which demonstrated its efficacy in eliciting significant skeletal muscle adaptations, including increased lean mass and improved insulin sensitivity, in T2DM models [23]. Throughout the intervention period, both the SED and RE groups continued to be fed a high-fat diet.

### 2.3. Glucose and Insulin Tolerance Tests

(1) Glucose tolerance tests (GTTs): After a 12 h overnight fast (from 20:00 to 08:00 the following day), FBG was measured. Mice were then intraperitoneally injected with glucose solution at a dose of 1.0 g/kg. Blood glucose levels were assessed at 15, 30, 60, 90 and 120 min after injection using a Roche blood glucometer (Berlin, Germany).

(2) Insulin tolerance tests (ITTs): Mice were transferred to clean cages and fasted for 6 h (8:00–14:00). Following baseline blood glucose measurement, insulin was administered intraperitoneally at 1 IU/kg. Blood glucose concentrations were measured at 15, 30, 60, 90, and 120 min post-injection.

### 2.4. Body Composition

The body composition of the mice, including fat content and lean mass, was assessed by an EchoMRI body composition analyzer (Echo Medical Systems, Houston, TX, USA).

### 2.5. Animal Sampling and Treatment

All mice were anesthetized by spontaneous inhalation of 3% isoflurane (Thermo Fisher Scientific, Waltham, MA, USA) 24 h after the last exercise training and subsequently euthanized by exsanguination. Body weight was recorded, and blood samples along with bilateral tibialis anterior (TA) muscles were collected. Serum was separated by centrifugation at 3000 rpm for 15 min. All harvested tissues were snap-frozen and stored at −80 °C for subsequent quantitative PCR (qPCR) and Western blot (WB) analyses.

### 2.6. Serum Biochemical Analysis

Fasting serum insulin levels were determined by using a mouse INS ELISA kit (CEA448Mu; Cloud-Clone Corp., Houston, TX, USA). The serum levels of high-density lipoprotein (HDL), low-density lipoprotein (LDL), triglycerides (TG), and total cholesterol (TC) were quantified using a biochemical analyzer in accordance with the manufacturer’s instructions.

### 2.7. Hematoxylin-Eosin and Sirius Red Staining

Hematoxylin and eosin (H&E) staining was performed on tissue sections according to the manufacturer’s protocol (Servicebio, Inc., Wuhan, China). Briefly, sections were stained with hematoxylin for 3 min, followed by differentiation in acid alcohol and bluing in ammonia water. After rinsing under running water for 60 min, the sections were immersed in distilled water and dehydrated through a graded ethanol series. Subsequently, they were counterstained with eosin for 30 s and rinsed with tap water. Following staining, the cytoplasm was specifically marked red. The slides were then mounted and examined under a Labophot-2 microscope (Nikon, Tokyo, Japan). Images were captured at 400× magnification and analyzed using ImageJ software (version 1.8.0, National Institutes of Health, Bethesda, MD, USA).

Frozen sections of TA muscle were stained using a 0.1% PicroSirius Red Stain Kit (ab15068, Abcam, Cambridge, UK). The stained sections were then imaged at 200× magnification. Collagen fibers were visualized in red, and the positively stained areas were quantified using ImageJ software.

### 2.8. Protein Extraction and Western Blotting

Protein extraction was performed according to a previously described protocol [23]. For Western Blotting, 40 μg of protein sample was separated by electrophoresis using 4–20% gradient SDS-PAGE gel (Bio-Rad Laboratories, Hercules, CA, USA) and subsequently transferred to a PVDF membrane. After transfer, the membrane was blocked with 5% non-fat milk and incubated overnight at 4 °C with primary antibodies against the following proteins: Protein kinase B (Akt), phosphorylated protein kinase B (p-Akt), cluster of differentiation 36 (CD36), carnitine palmitoyl transferase-1α (CPT-1α), dynamin-related protein 1 (DRP1), fission protein 1 (FIS1), mammalian target of rapamycin (mTOR), phosphorylated mammalian target of rapamycin (p-mTOR), Muscle Atrophy F-box (Atrogin-1), Muscle RING-finger 1 (MuRF1), nuclear respiratory factor 2 (NRF2), ribosomal protein 70S6 kinase (p70S6K), phosphorylated ribosomal protein 70 S6 kinase (p-p70S6K), peroxisome proliferator activate receptors α (PPARα), pyruvate dehydrogenase kinase 4 (PDK4), Phosphatidylinositol 3-kinase (PI3K), peroxisome proliferator activated receptor gamma coactivator-1α (PGC-1α), 4E-binding protein 1 (4EBP1), phosphorylated 4E-binding protein 1 (p-4EBP1), fibroblast growth factor 21 (FGF21) and GAPDH. Following three washes with TBST buffer, the membrane was incubated with an HRP-conjugated secondary antibody. Protein bands were visualized using an enhanced chemiluminescence (ECL) detection system (Thermo Scientific, Waltham, MA, USA) and quantified with ImageJ software (version 1.8.0, National Institutes of Health, Bethesda, MD, USA). GAPDH was used as the internal loading control for normalization.

### 2.9. Quantitative Real-Time PCR Analysis

Total RNA was extracted from the TA muscle of mice in each group using TRIzol reagent (9101,Takara Bio Inc., Shiga, Japan). Subsequently, an equal amount of RNA was reverse transcribed into cDNA using a commercial reverse transcription kit (RR036A, Takara Bio Inc., Shiga, Japan) following the manufacturer’s instructions. Quantitative real-time PCR (qPCR) was performed on the QuantStudio™ 3 system (Thermo Fisher Scientific, Waltham, MA, USA). The amplification protocol consisted of an initial denaturation step at 95 °C for 5 min, followed by 40 cycles of denaturation at 95 °C for 10 s and annealing/extension at 60 °C for 30 s. Gene expression levels were normalized to *β-actin*, and the relative quantification was calculated using the 2^−∆∆Ct^ method. The sequences of all primers used are listed in Table 1.

### 2.10. Statistical Analysis

All statistical analyses were performed using Graphpad Prism version 8.0 (GraphPad Software, USA). Data are presented as mean ± standard deviation. Group means were compared using one-way analysis of variance (ANOVA) followed by Tukey’s post hoc test. Differences were considered statistically significant at *p* < 0.05 and highly significant at *p* < 0.01.

## 3. Results

### 3.1. RE Improves Body Composition and Metabolic Indexes of T2DM Mice

**Figure 1:**
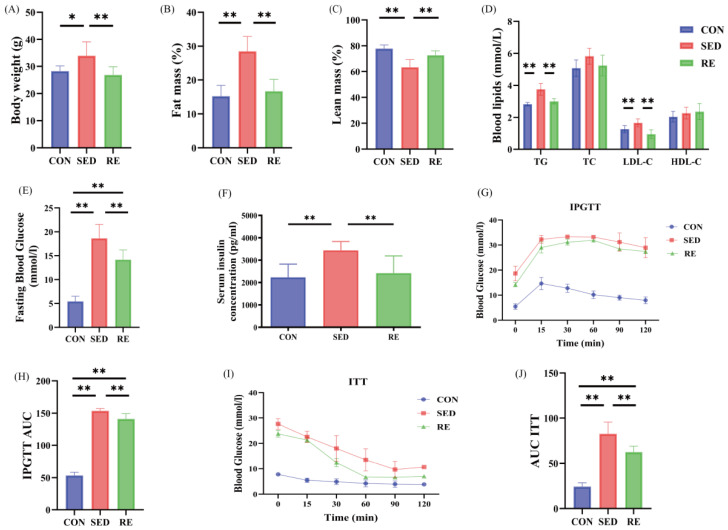
RE improves body composition and metabolic indexes of T2DM mice. (**A**) Body weight; (**B**) Fat mass (%); (**C**) Lean mass (%); (**D**) Lipid Profile; (**E**) Fasting blood glucose; (**F**) Serum insulin concentration; (**G**,**H**) Blood glucose levels and their AUC at 15, 30, 60, 90, and 120 min after glucose injection (IPGTT, 1 g/kg BW) in mice fasted overnight; (**I**,**J**) Blood glucose levels and their AUC at 0, 15, 30, 60, 90, and 120 min after insulin injection (ITT, 1IU/kg BW) in mice fasted for 6 h. All data are expressed as mean ± SD, *n* = 8 per group, * *p* < 0.05, ** *p* < 0.01.

T2DM mouse model was established using a 12-week HFD combined with intraperitoneal injection of STZ followed by an 8-week of RE intervention in the RE group. The results showed that body weight (Figure 1A) and fat mass (Figure 1B) were significantly higher in the SED group compared to the CON group. In contrast, both body weight and fat mass were significantly reduced in the RE group relative to the SED group. Furthermore, lean mass was significantly reduced in T2DM mice compared with the control group, but was significantly increased following RE training (Figure 1C). These findings indicate that RE improves body composition in T2DM mice.

We further evaluated changes in blood lipid in the experimental mice. The results revealed that compared to the CON group, serum levels of TG and LDL-C were significantly increased in the SED group, RE markedly reduced these levels in the T2DM mice. However, no significant changes were observed in TC, HDL-C, and following RE intervention (Figure 1D). Additionally, FBG and serum insulin concentration were significantly higher in the SED group than in the CON group. RE led to a significant reduction in both FBG (Figure 1E) and serum insulin levels (Figure 1F) in the T2DM mice. Furthermore, IPGTT and ITT were performed. Quantitative analysis of the total area under the curve (AUC) indicated that mice in the SED group exhibited impaired glucose tolerance and insulin sensitivity compared to those in the CON group. In contrast, RE treatment improved both glucose tolerance (Figure 1H) and insulin sensitivity (Figure 1J) relative to the SED group. Taken together, these findings demonstrate that RE induces significant improvements in body composition and metabolic parameters in T2DM mice.

### 3.2. RE Counteracts Skeletal Muscle Atrophy in T2DM Mice

**Figure 2:**
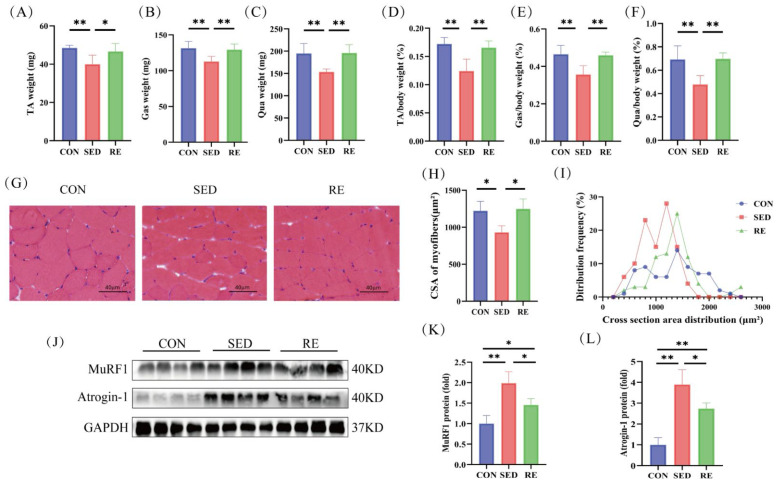
RE counteracts skeletal muscle atrophy in T2DM mice. (**A**–**C**) Absolute weight of TA, Gas and Qua (*n* = 8 per group); (**D**–**F**) Ratio of TA, Gas and Qua to BW (*n* = 8 per group); (**G**) Representative image of hematoxylin and eosin (HE) staining of TA muscle fibers (magnification ×400), (*n* = 3 per group); (**H**) Quantified muscle cell fiber cross-sectional area in T2DM mice after RE treatment; (**I**) Percentage distribution of muscle fiber CSA in mice; (**J**) Western blot analysis showed MuRF1 and Atrogin-1 protein levels in skeletal muscle (*n* = 4 per group); (**K**,**L**) Quantification of MuRF1 and Atrogin-1 expression levels shown in (**J**). All data are expressed as mean ± SD, * *p* < 0.05, ** *p* < 0.01. Original images can be found at Supplementary Materials.

To investigate the effect of RE on muscle atrophy in T2DM mice, we first assessed the absolute weight of skeletal muscle (tibialis anterior, gastrocnemius, and quadriceps). A significant decrease in absolute muscle mass was observed in SED mice compared to the CON group, and this loss was effectively rescued by RE intervention (Figure 2A–C). To address the potential confounding effect of overall body weight changes, we also calculated the relative weight normalized to body weight. Consistent with the absolute mass data, the ratios of TA/BW (Figure 2D), Gas/BW (Figure 2E), and Qua/BW (Figure 2F) were significantly lower in SED mice and elevated by RE. To provide direct evidence of muscle atrophy beyond relative weight changes, we performed histological analysis of muscle sections. The cross-sectional area (CSA) of myofibers, a gold-standard indicator of atrophy, was significantly reduced in SED mice compared to CON mice (Figure 2G). Notably, RE treatment markedly rescued the CSA (Figure 2H). Furthermore, the frequency distribution of myofiber size confirmed a leftward shift (towards smaller sizes) in SED mice, which was reversed by RE (Figure 2I). At the molecular level, the protein expression of two key atrophy-related E3 ubiquitin ligases, MuRF1 and Atrogin-1, was significantly upregulated in the skeletal muscle of SED mice, confirming the activation of the ubiquitin-proteasome pathway. RE treatment effectively suppressed this increase (Figure 2J–L). Together, these multi-faceted findings—from absolute mass, morphology, to molecular markers—converge to demonstrate that RE effectively attenuates true skeletal muscle atrophy in T2DM mice, independent of body weight considerations.

### 3.3. RE Alleviates Fibrosis and Inflammation of Skeletal Muscle in T2DM Mice

**Figure 3:**
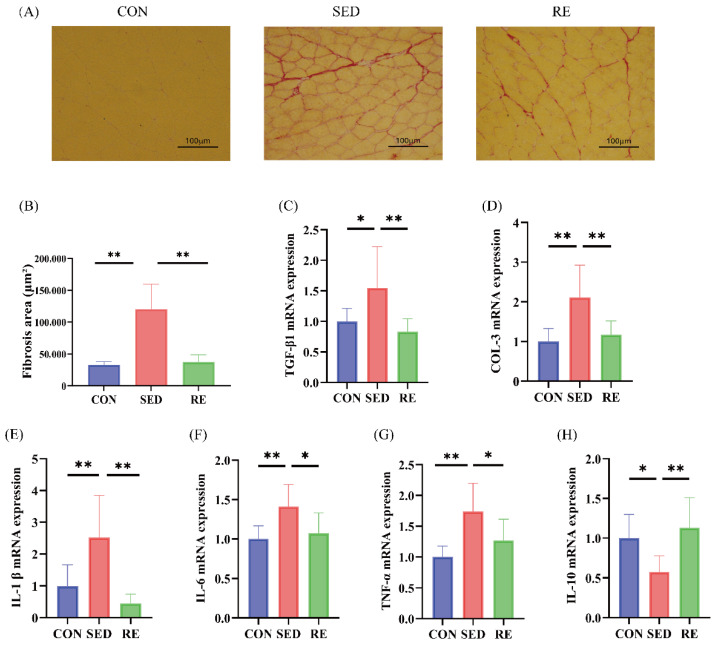
RE improves fibrosis and inflammation of skeletal muscle in T2DM mice. (**A**) Sirius red staining of skeletal muscle (magnification ×200), (*n* = 3 per group); (**B**) Quantification of the fibrosis area; (**C**–**H**) mRNA expression levels of *TGF-β1*, *COL-3*, *IL-1β*, *IL-6*, *TNF-α*, and *IL-10* (*n* = 8 per group). All data are expressed as mean ± SD, * *p* < 0.05, ** *p* < 0.01.

Sirius red staining was used to evaluate the extent of tissue fibrosis. The results revealed a significant increase in the fibrotic area in the SED group compared to the CON group, which was markedly attenuated following RE intervention (Figure 3A,B). To further characterize fibrosis at the molecular level, we examined the expression of fibrosis-related markers, transforming growth factor-beta 1 (*TGF-β1*) and collagen type III (*COL-3*), via RT-qPCR. The mRNA levels of both *TGF-β1* and *COL-3* were significantly elevated in the SED group compared to controls. In contrast, RE treatment significantly reduced the expression of these genes relative to the SED group (Figure 3C,D).

Chronic inflammation is not only an initiating factor in tissue fibrosis [33], but also plays a critical role in the pathogenesis of T2DM [34]. Accordingly, we assessed the mRNA expression levels of both pro-inflammatory (*IL-1β*, *IL-6*, and *TNF-α*) and anti-inflammatory (*IL-10*) cytokines in the skeletal muscle across experimental groups. The results demonstrated that mRNA expression of *TNF-α*, *IL-1β*, and *IL-6* was significantly upregulated in the SED group, while *IL-10* expression was downregulated compared to the CON group. Following an 8-week RE intervention, mRNA levels of *IL-10* were significantly increased, whereas those of the pro-inflammatory cytokines were markedly reduced (Figure 3E–H). These findings indicate that RE effectively attenuates skeletal muscle inflammation in T2DM mice.

### 3.4. RE Activates FGF21/PI3K/Akt Signaling Pathway and Promotes Skeletal Muscle Protein Synthesis in T2DM Mice

**Figure 4:**
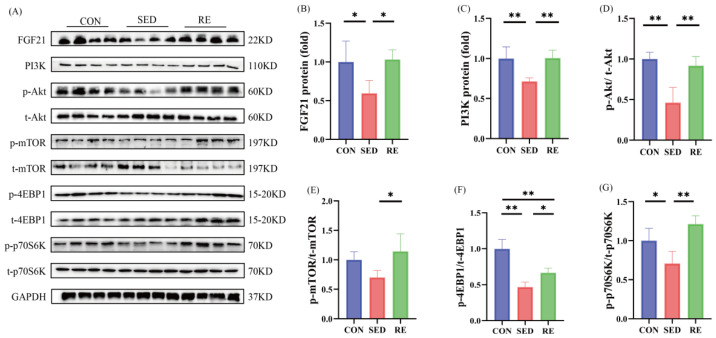
RE activates FGF21/PI3K/Akt signaling pathway and promotes skeletal muscle protein synthesis in T2DM mice. (**A**) Western blot results of FGF21, PI3K, p-Akt, t-Akt, p-mTOR, t-mTOR, p-4EBP1, t-4EBP1, p-p70S6K, t-p70S6K and GAPDH in skeletal muscle (*n* = 4 per group); (**B**–**G**) protein expression levels of FGF21, PI3K, p-Akt/t-Akt, p-mTOR/mTOR, p-4EBP1/t-4EBP1, p-p70S6K/t- p70S6K in skeletal muscle (*n* = 4 per group). All data are expressed as mean ± SD, * *p* < 0.05, ** *p* < 0.01. Original images can be found at Supplementary Materials.

Previous studies have shown that FGF21 ameliorates metabolic diseases through activation of the PI3K/Akt signaling pathway [35,36]. To investigate whether RE influences FGF21 expression and PI3K/Akt signaling in skeletal muscle, we evaluated the protein levels of key components within this pathway. As shown in Figure 4A–D, the protein expression of FGF21, PI3K, and p-Akt/t-Akt were significantly downregulated in the skeletal muscle of SED mice compared to the CON group. In contrast, RE intervention significantly upregulated the expression of FGF21, PI3K, and p-Akt/t-Akt compared to the SED group. mTOR, a downstream effector of Akt, promotes protein synthesis by phosphorylating downstream targets including p70S6K and 4EBP1. Our results showed that phosphorylation levels of 4EBP1 (p-4EBP1/t-4EBP1)and p70S6K (p-p70S6K/t-p70S6K) were significantly reduced in SED mice relative to controls (Figure 4F,G). However, 8 weeks of RE significantly enhanced the phosphorylation levels of mTOR (p-mTOR/mTOR), 4EBP1, and p70S6K in the skeletal muscle of T2DM mice (Figure 4A,E–G). Collectively, these results suggest that RE upregulates FGF21 expression and activates the PI3K/Akt signaling pathway, thereby promoting protein synthesis in the skeletal muscle of T2DM mice.

### 3.5. RE Improves Glycolipid Metabolism Disorder in Skeletal Muscle of T2DM Mice

**Figure 5:**
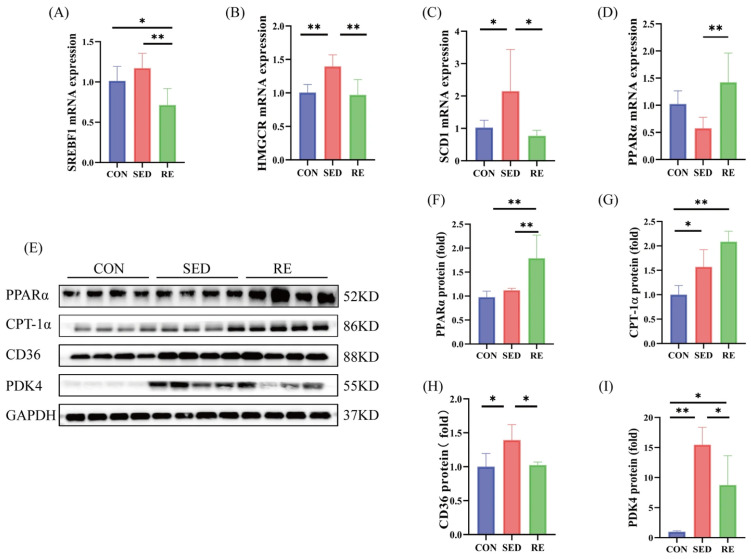
RE improves glycolipid metabolism disorder in skeletal muscle of T2DM mice. (**A**–**D**) mRNA expression levels of *SREBF1, HMGCR, SCD1* and *PPARα* (*n* = 8 per group); (**E**) Western blot results of PPARα, CPT-1α, CD36 and PDK4 (*n* = 4 per group); (**F**–**I**) protein expression levels of PPARα, CPT-1α, CD36 and PDK4 (*n* = 4 per group). All data are expressed as mean ± SD, * *p* < 0.05, ** *p* < 0.01. Original images can be found at Supplementary Materials.

To evaluate the effect of RE on skeletal muscle glycolipid metabolism in T2DM mice, we detected the mRNA and protein expression of key factors involved in glycolipid metabolism. Among these, *SREBF1*, *HMGCR*, and *SCD1* are associated with lipid synthesis. As shown in (Figure 5A–C), the mRNA expression levels of *HMGCR* and *SCD1* were significantly upregulated in the skeletal muscle of SED mice compared to the CON group, although no significant change was observed in *SREBF1* expression. After RE intervention, the mRNA expression of *SREBF1*, *HMGCR*, and *SCD1* was significantly reduced in the skeletal muscle of T2DM mice. These results suggest that RE suppresses lipid synthesis in the skeletal muscle of T2DM mice.

We examined the expression of key factors involved in lipid oxidation and transport. No significant differences were observed in the mRNA or protein expression of *PPARα*in the skeletal muscle of SED mice compared to the CON mice. In contrast, RE significantly promoted *PPARα* mRNA and protein expression in skeletal muscle of T2DM mice (Figure 5D–F). In addition, protein levels of CPT-1α and CD36 were significantly elevated in the SED group relative to controls. Following RE intervention, CD36 protein expression was markedly reduced, while CPT-1α protein levels remained unchanged (Figure 5E,G,H). We also examined the protein expression of glucose metabolism markers. PDK4 expression was significantly higher in the SED group than in the control group, and was significantly decreased after RE (Figure 5E,I). These results suggest that RE enhances lipid oxidation and promotes glucose oxidation in the skeletal muscle of T2DM mice.

### 3.6. RE Improves Mitochondrial Biogenesis and Dynamics in Skeletal Muscle of T2DM Mice

**Figure 6:**
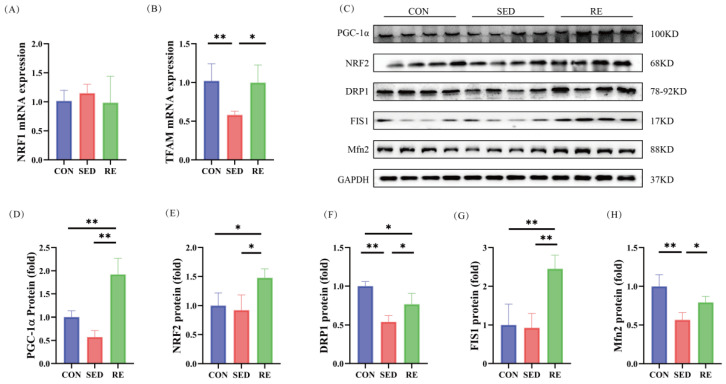
RE improves mitochondrial biosynthesis and dynamics in skeletal muscle of T2DM mice. (**A**,**B**) mRNA expression of *NRF1* and *TFAM* (*n* = 8 per group); (**C**) Western blot results of PGC-1α, NRF2, DRP1, FIS1, Mfn2 and GAPDH (*n* = 4 per group); (**D**–**H**) protein expression levels of PGC-1α, NRF2, DRP1, FIS1, Mfn2 (*n* = 4 per group). All data are expressed as mean ± SD, * *p* < 0.05, ** *p* < 0.01. Original images can be found at Supplementary Materials.

PGC-1α, *NRF1*, NRF2, and *TFAM* are key regulators of mitochondrial biogenesis. No significant differences in *NRF1* mRNA expression were observed among the three groups (Figure 6A). However, *TFAM* mRNA expression was significantly lower in the skeletal muscle of SED mice compared to the CON group, and RE significantly upregulated *TFAM* expression (Figure 6B). At the protein level, PGC-1α and NRF2 expression in the SED group did not differ significantly from the CON group. Nevertheless, 8 weeks of RE significantly increased the protein level of both PGC-1α and NRF2 in T2DM mice (Figure 6C–E). These results indicate that RE enhances mitochondrial biogenesis in the skeletal muscle of T2DM mice.

Mitochondrial fission is essential for maintaining mitochondrial function. We evaluated the protein expression of DRP1, FIS1, and Mfn2. Compared with the CON group, the SED group exhibited significantly reduced protein expression of DRP1 and Mfn2, while FIS1 levels remained unchanged. RE significantly upregulated the expression of DRP1, FIS1, and Mfn2 (Figure 6C,F–H), suggesting that RE promotes both mitochondrial fission and fusion processes in the skeletal muscle of T2DM mice.

## 4. Discussion

T2DM is a metabolic disorder characterized by persistent hyperglycemia and endocrine dysregulation. Previous animal studies have indicated that 8 weeks of RE can reduce FBG and improve glucose tolerance in Zucker diabetic rats [37], a finding corroborated by human clinical studies [38,39]. Consistent with these reports, our study demonstrated that 8 weeks of RE significantly reduced FBG and serum insulin levels, while also enhancing glucose and insulin tolerance in T2DM mice, indicating that RE contributes to improved glucose homeostasis. Additionally, T2DM is commonly associated with disordered lipid metabolism. Clinical evidence suggests that RE significantly lowers TG, LDL-C, and TC in T2DM patients, without markedly affecting HDL-C levels [40,41]. In the present study, RE significantly reduced serum TC and LDL-C in T2DM mice but did not significantly alter TG and HDL-C levels. These discrepancies may be attributable to interspecies differences between animal models and humans. Moreover, diminished muscle mass impairs glucose uptake into skeletal muscle, resulting in sustained hyperglycemia and further compromising skeletal muscle function [42]. Therefore, increasing lean mass remains a critical objective in T2DM management for patients. Existing studies have established RE as an effective intervention for reducing body mass index (BMI) and body fat percentage, while increasing lean mass in individuals with T2DM [43,44]. Our findings align with these results, showing that RE reduces fat mass and increases lean mass in T2DM mice. In conclusion, these findings underscore the importance of RE as a key component in the comprehensive management of T2DM. More importantly, the systemic metabolic improvements are underpinned by a fundamental restoration of insulin signaling within the skeletal muscle. Our data reveal that RE significantly activates the PI3K/Akt pathway, a central signaling cascade for insulin-mediated glucose uptake and metabolism. The activation of this pathway directly addresses the core defect of insulin resistance in T2DM, suggesting that the primary metabolic impact of RE is to enhance skeletal muscle insulin sensitivity and local metabolic function. This is of critical importance, as skeletal muscle is a major site for glucose disposal. Taken together, these findings underscore the importance of RE as a key component in the comprehensive management of T2DM. While the reduction in systemic fasting blood glucose and the improvement in glucose tolerance observed in our study were significant yet modest, our data point to a more profound metabolic restoration at the tissue level. The activation of the FGF21/PI3K/Akt signaling pathway (Section 3.4) represents a key mechanism by which RE improves skeletal muscle insulin sensitivity. The improvement in muscle metabolic health likely serves as the foundation for the subsequent, more generalized metabolic amelioration observed at the systemic level.

Skeletal muscle atrophy, characterized by reduced muscle mass and decreased CSA of muscle fibers, is a common pathological feature of T2DM. Our results confirm that T2DM leads to a loss of skeletal muscle mass, as indicated by significantly lower muscle-to-body weight ratios of the TA, Gas and Qua, along with reductions in the absolute weights of these muscles and a decrease in muscle fiber CSA, consistent with previous reports [45,46]. Current clinical guidelines recommend RE as the primary intervention for sarcopenia [4]. Numerous animal studies have further demonstrated that RE not only counteracts skeletal muscle atrophy induced by aging [47], myocardial infarction [10], and Alzheimer’s disease [48], but also promotes muscle hypertrophy. In line with these findings, our study found that RE significantly increased the relative muscle weight (expressed as the ratios of TA, Qua, and Gas to body weight) and the absolute weights of these muscles. Moreover, RE intervention enhanced the CSA of muscle fibers in the TA of T2DM mice. This improvement in muscle fiber CSA is of particular relevance, as previous studies have established a strong correlation between increased CSA and enhancements in muscle strength and functional capacity [49,50]. MuRF1 and Atrogin-1, key regulators of protein degradation, are established markers of skeletal muscle atrophy. Both human and animal studies have reported elevated expression levels of MuRF1 and Atrogin-1 in T2DM, where they correlate strongly with muscle wasting [51,52]. Inhibition of these genes has been shown to reduce protein degradation and attenuate muscle atrophy in T2DM mice induced by a high-fat diet and STZ [53]. Previous research also indicates that RE attenuates skeletal muscle atrophy following myocardial infarction [10] and disuse [54] by suppressing MuRF1 and Atrogin-1 expression. In this study, we observed significantly upregulated MuRF1 and Atrogin-1 levels in the skeletal muscle of T2DM mice, which were markedly downregulated after RE intervention. Collectively, evidence from absolute mass, morphology, and molecular markers demonstrates that RE effectively mitigates true skeletal muscle atrophy in T2DM mice, independent of body weight.

FGF21 is a key metabolic regulator [55] that also plays an essential role in the maintenance of muscle mass [56]. Previous studies have reported significantly reduced FGF21 expression in the skeletal muscle of T2DM and IR rats [15], while FGF21 knockout exacerbated skeletal muscle atrophy and IR in obese mice [16,17]. Consistent with these findings, the current study observed markedly decreased FGF21 expression in the skeletal muscle of T2DM mice, further supporting the important role of FGF21 in T2DM- and obesity-induced skeletal muscle atrophy. Moreover, FGF21 has been shown to exert protective effects across multiple tissues and organ through regulation of the PI3K/Akt signaling pathway [57,58]. In T2DM, skeletal muscle energy metabolism is often impaired, leading to suppressed PI3K/Akt pathway activity and reduced protein synthesis [59]. Our previous work also demonstrated decreased ratios of p-PI3K/PI3K and p-Akt/Akt in the skeletal muscle of T2DM mice [23]. Here, we found that protein levels of FGF21, PI3K, and the p-Akt/Akt ratio were significantly reduced in the skeletal muscle of T2DM mice, indicating that T2DM inhibited the FGF21/PI3K/Akt pathway. FGF21 is also recognized as an exerkines. Previous research indicates that RE significantly promotes FGF21 expression in the soleus muscle of obese mice, correlating strongly with gains in muscle strength [60]. In the present study, RE significantly increased the protein expression of FGF21, PI3K, and p-Akt/Akt, suggesting that RE may mitigate skeletal muscle atrophy in T2DM mice by upregulating FGF21 and activating the PI3K/Akt pathway. Furthermore, it is well established that mTOR, a major downstream effector of PI3K/Akt, serves as a central regulator of protein synthesis. The activation of its downstream targets, p70S6K1 and 4EBP1, is critical for the regulation of skeletal muscle fiber size [61]. Previous studies have reported inhibited mTORC1 activity along with reduced phosphorylation of p70S6K and 4EBP in the skeletal muscle of both T2DM patients [62] and T2DM rats [13], leading to diminished protein synthesis. Our results also showed significantly decreased ratios of p-p70S6K/t-p70S6K and p-4EBP1/t-4EBP1 in T2DM mice, which were markedly elevated following RE. RE also significantly promoted the protein levels of p-mTOR/t-mTOR. These findings imply that RE may enhance protein synthesis and mitigate skeletal muscle atrophy in T2DM mice through activation of the FGF21/PI3K/Akt/mTOR signaling axis.

Skeletal muscle fibrosis, characterized by excessive deposition of extracellular matrix (particularly collagen), is a hallmark of muscle atrophy that compromises muscle function and impairs regeneration capacity [33,63]. Consistent with clinical and experimental observations [64,65], we found significantly elevated mRNA expression of *TGF-β1* and *COL-3*, and fibrosis area increased in T2DM mice, further corroborating the role of T2DM in promoting skeletal muscle fibrosis. Although aerobic exercise has been shown to attenuate fibrosis in the gastrocnemius muscle of db/db mice via inhibition of TGF-β signaling and reduction in type I collagen [66]. Our results demonstrate that RE similarly reduces fibrotic area and downregulates *TGF-β1* and *COL-3* expression in T2DM mice, highlighting its therapeutic potential against T2DM-induced muscle fibrosis. Fibrosis commonly arises from chronic inflammation, a key feature of T2DM pathophysiology, wherein aberrant cytokine secretion disrupts extracellular matrix homeostasis [5,63,67]. In line with previous reports [68,69], we observed elevated levels of pro-inflammatory cytokines (*TNF-α*, *IL-1β*, *IL-6*) and reduced *IL-10* in T2DM skeletal muscle. Exercise is a well-established intervention for alleviating skeletal muscle inflammation. For instance, swimming exercise reduced *IL-6* and *TNF-α* in the skeletal muscle of high-fat diet-fed rats [70]. Aerobic exercise downregulated IL-1β and upregulated IL-10 in the skeletal muscle of T2DM mice, and RE was reported to lower IL-6 in T2DM rats, albeit without significant effects on TNF-α or IL-1β [71]. In our study, an 8-week RE intervention significantly suppressed *TNF-α*, *IL-1β*, and *IL-6* mRNA expression while enhancing *IL-10* levels. The broader anti-inflammatory efficacy observed here may be attributable to the longer duration of exercise training. Previous studies indicate that genetic ablation of FGF21 exacerbates skeletal muscle inflammation and insulin resistance in obese mice [16,17], while activation of the PI3K/Akt pathway exerts anti-inflammatory and anti-fibrotic effects [21,72,73], including downregulation of TGF-β1 and COL-3 expression and amelioration of skeletal muscle fibrosis [22]. Our results indicate that RE activates the FGF21/PI3K/AKT signaling pathway. Therefore, these findings suggest that RE attenuates inflammation and fibrosis in T2DM mice—at least partially—through activation of the FGF21/PI3K/Akt pathway, ultimately alleviating skeletal muscle atrophy.

Our previous study revealed significant lipid accumulation in the skeletal muscle of T2DM mice [74], which impairs insulin-mediated glucose uptake and exacerbates muscle atrophy [75]. CD36, a key regulator of lipid metabolism, facilitates long-chain fatty acid uptake [76]. Consistent with human and animal studies demonstrating that a high-fat diet upregulates CD36 expression in skeletal muscle [77,78], we observed markedly elevated CD36 levels in the skeletal muscle of T2DM mice, indicating enhanced fatty acid uptake. Smith et al. reported that 8 weeks of aerobic exercise effectively suppressed CD36 protein expression and reduced lipid accumulation in skeletal muscle of Zucker rats [79]. Similarly, our results demonstrate that 8 weeks of RE significantly reduced CD36 expression in skeletal muscle of T2DM mice, suggesting that RE may attenuate lipid deposition by limiting fatty acid uptake. In addition, PPARα and CPT-1α play pivotal roles in fatty acid oxidation. We found that RE significantly upregulated both CPT-1α and PPARα expression while suppressing the mRNA levels of lipogenic genes (*HMGCR*, *SREBF1*, and *SCD1*), indicating that RE enhances lipid oxidation and inhibits lipid synthesis in skeletal muscle of T2DM mice. Previous studies have shown that activation of the PI3K/Akt/PPARα/CPT-1α pathway improved glucose and lipid metabolism in db/db mice [80]. Han et al. further demonstrated that PI3K/Akt pathway reduces lipid synthesis and accumulation by downregulating *SCD1* and *SREBP-1c* (encoded by *SREBF1*), thereby alleviating high-fat diet-induced atrophy in the gastrocnemius muscle of obese mice [81]. Thus, RE may improve disordered lipid metabolism and counteract skeletal muscle atrophy in T2DM mice via upregulation of FGF21/PI3K/Akt signaling pathway. Furthermore, PDK4 is an important regulator of glucose metabolism. Elevated PDK4 expression has been linked to skeletal muscle atrophy in conditions such as amyotrophic lateral sclerosis [82] and cancer cachexia [83]. We observed significantly increased PDK4 protein expression in skeletal muscle of T2DM mice, consistent with earlier animal studies [84]. Yang et al. showed that RE effectively inhibited PDK4 levels in human skeletal muscle [85]. In line with this, our results show that RE significantly reduced PDK4 expression in skeletal muscle of T2DM mice, suggesting enhanced glucose utilization. Since the PI3K/Akt pathway negatively regulates PDK4 expression [86], RE may further ameliorate skeletal muscle atrophy in T2DM mice by activating FGF21/PI3K/Akt pathway and improving glucose utilization.

Disorder of glucose and lipid metabolism, along with insulin resistance, can contribute to mitochondrial dysfunction in skeletal muscle. Mitochondrial quality control (including biogenesis and dynamics) is essential for maintaining mitochondrial homeostasis and preserving muscle mass [87]. Mitochondrial biogenesis is coregulated by PGC-1α and several transcription factors, including NRF1, NRF2, and TFAM. Animal studies have revealed significantly reduced expression of PGC-1α in the skeletal muscle of T2DM mice [88] and rats [89]. Human studies further indicate decreased levels of NRF2, PGC-1α, and TFAM in skeletal muscle from T2DM patients [90], although NRF1 expression remains largely unchanged [91]. Transcriptomic analysis of muscle biopsies from individuals with sarcopenia also shows reduced mitochondrial content and diminished PGC-1α/ERRα signaling [92], underscoring the link between mitochondrial quantity and biogenesis. Our previous study showed that high-intensity interval training effectively promoted mitochondrial biogenesis in skeletal muscle of T2DM mice [74]. While Theilen et al. reported that exercise mitigates muscle atrophy by activating the PGC-1α-NRF1/2-TFAM pathway [93]. In line with these findings, we found that RE significantly upregulates the expression of PGC-1α, NRF2, and *TFAM*, indicating that RE promotes mitochondrial biogenesis and attenuates skeletal muscle atrophy in T2DM mice. Mitochondrial health also depends on balanced fusion and fission processes; disruption of these dynamics is associated with various pathologies, including skeletal muscle atrophy [94,95]. Studies have shown reduced expression of Mfn2, DRP1, and FIS1 in T2DM-induced muscle atrophy [96,97]. Our study found that DRP1 and Mfn2 protein expression was significantly reduced in the skeletal muscle of T2DM mice, but FIS1 expression did not change significantly. Similarly, Kruse et al. demonstrated that FIS1 expression in skeletal muscle was not altered in patients with T2DM [98]. This may be because FIS1 is not the only player directing DRP1 to the mitochondrial outer membrane [99]. Notably, overexpression of Mfn2 promotes mild muscle hypertrophy in aged mice [100], and elevated DRP1 and FIS1 expression alleviates muscle atrophy in Zucker rats [96], highlighting the importance of mitochondrial dynamics in maintaining muscle integrity. Our results align with previous studies demonstrating that RE increases Mfn2, DRP1, and FIS1 expression in skeletal muscle in both healthy [101] and T2DM mice [23], suggesting that RE improves T2DM-related muscle atrophy by enhancing mitochondrial fusion and fission. Moreover, activation of PI3K/Akt pathway has been shown to confer protection in diabetic cardiomyopathy [102] and diabetic tubulopathy [103] by regulating mitochondrial biogenesis and dynamics. Given that FGF21 can directly activate the PI3K/Akt pathway, the present findings indicate that RE may restore impaired mitochondrial biogenesis and dynamics in the skeletal muscle of T2DM mice via the FGF21/PI3K/Akt signaling axis, ultimately ameliorating muscle atrophy.

While this study provides compelling evidence for the beneficial effects of RE on diabetic sarcopenia via the FGF21/PI3K/Akt pathway, several limitations should be acknowledged. Firstly, the findings are derived from a rodent model of T2DM. Although this widely utilized, the pathophysiological differences between STZ-induced diabetic mice and human T2DM may affect the direct translatability of the results. Secondly, while the upregulation of FGF21 is documented, the study does not include intervention experiments using FGF21 inhibitors or knockout models to conclusively prove the essential role of FGF21 in mediating the observed effects of RE. Future research employing clinical trials and targeted mechanistic interventions is warranted to confirm and extend these findings. Finally, we acknowledge that the assessment of muscle function, such as direct measurements of contractile force, was not included in the present study. Although the observed improvements in muscle mass and morphology are strongly indicative of functional recovery, future work incorporating direct functional assays (e.g., grip strength, ex vivo contractility) will be valuable to fully establish the functional benefits of the intervention.

## 5. Conclusions

In conclusion, the present study demonstrates that an 8-week RE regimen counteracts skeletal muscle atrophy in T2DM mice by activating the FGF21/PI3K/Akt signaling pathway. This activation promoted protein synthesis, improved glycolipid metabolism and mitochondrial quality control, and reduced fibrosis and inflammation. Importantly, our findings reveal that RE mitigates diabetic sarcopenia through a multi-pathway integrative mechanism mediated by FGF21, thereby providing a novel molecular target and a theoretical foundation for exercise-based interventions against diabetic sarcopenia.

## Figures and Tables

**Table 1:** Quantitative real-time PCR primer sequence list.

Gene Name		Sequences
*β-actin*	Forward Reverse	ATCACTATTGGCAACGAGCGGTTC CAGCACTGTGTTGGCATAGAGGTC
*HMGCR*	Forward Reverse	GACCAACCTTCTACCTCAGCAAGC CCAGCCATCACAGTGCCACATAC
*SCD1*	Forward Reverse	AGCCTGTTCGTTAGCACCTTCTT GGTGTGGTGGTAGTTGTGGAAGC
*SREBF1*	Forward Reverse	CGACATCGAAGACATGCTTCAG GGAAGGCTTCAAGAGAGGAGC
*PPARα*	Forward Reverse	ACGATGCTGTCCTCCTTGATGAAC GATGTCACAGAACGGCTTCCTCAG
*TFAM*	Forward Reverse	GGAATGTGGAGCGTGCTAAAA TGCTGGAAAAACACTTCGGAATA
*NRF1*	Forward Reverse	GTTGCCCAAGTGAATTACTCTG TCGTCTGGATGGTCATTTCAC
*TGF-β1*	Forward Reverse	TGCGCTTGCAGAGATTAAAA CGTCAAAAGACAGCCACTCA
*COL-3*	Forward Reverse	GTTCACGTACACTGCCCTGA AAGGCGTGAGGTCTTCTGTG
*IL-1β*	Forward Reverse	GAAATGCCACCTTTTGACAGTG TGGATGCTCTCATCAGGACAG
*IL-6*	Forward Reverse	CAGCCACTGCCTTCCCTACT CAGTGCATCAT CGCTGTTCAT
*TNF-α*	Forward Reverse	CTTCTGTCTACTGAACTTCGGG CACTTGGTGGTTTGCTACGAC
*IL-10*	Forward Reverse	CAAGGAGCATTTGAATTCCC GGCCTTGTAGACACCTTGGTC

## Data Availability

The data that support the findings of this study are available from the corresponding author upon reasonable request due to privacy or ethical restrictions.

## Supplementary Materials

The following supporting information can be downloaded at: https://www.mdpi.com/article/10.3390/biom16010003/s1, File S1. Original Images for Western Blots.
