# Supplementary material for: Resistance Exercise Counteracts Skeletal Muscle Atrophy in T2DM Mice by Upregulating FGF21 and Activating PI3K/Akt Pathway
Source: Biomolecules. 2025 Dec 19;16(1):3. doi: 10.3390/biom16010003 (PMC12838593; doi:10.3390/biom16010003)

Figure 2

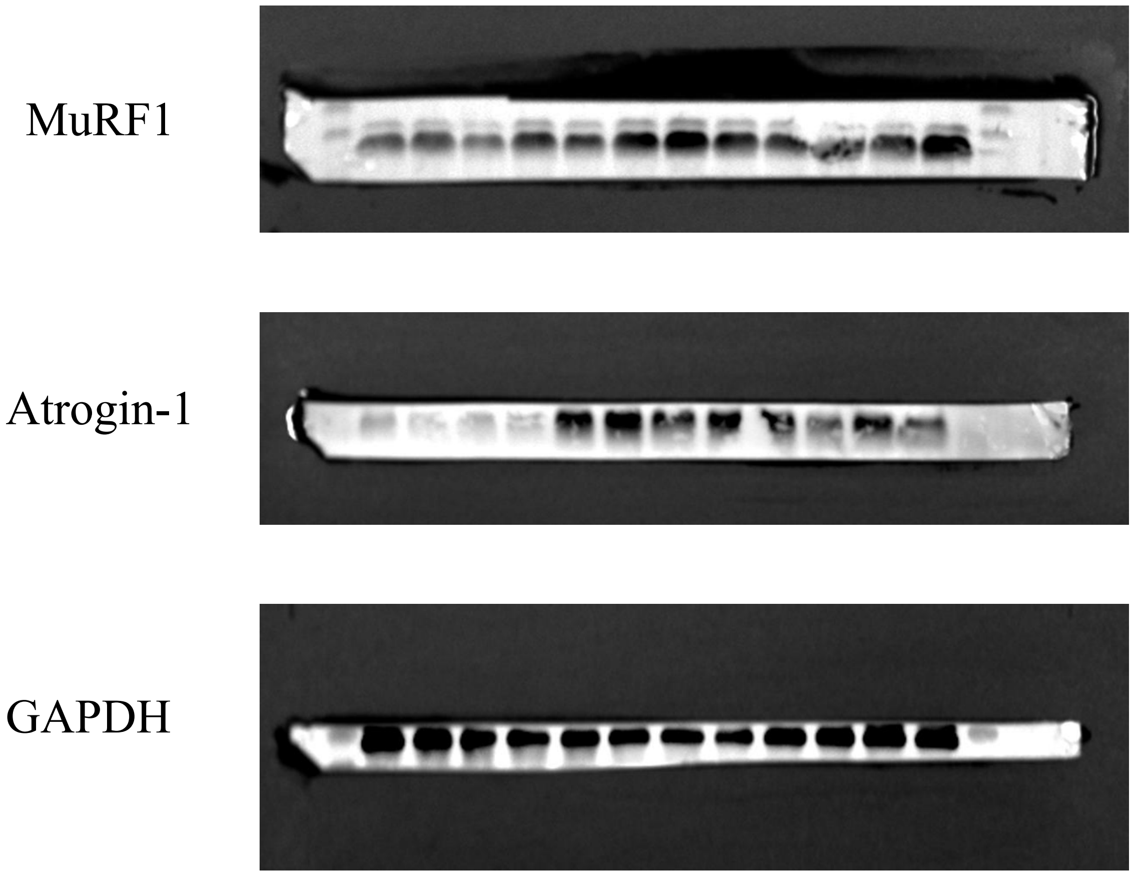

Figure 4

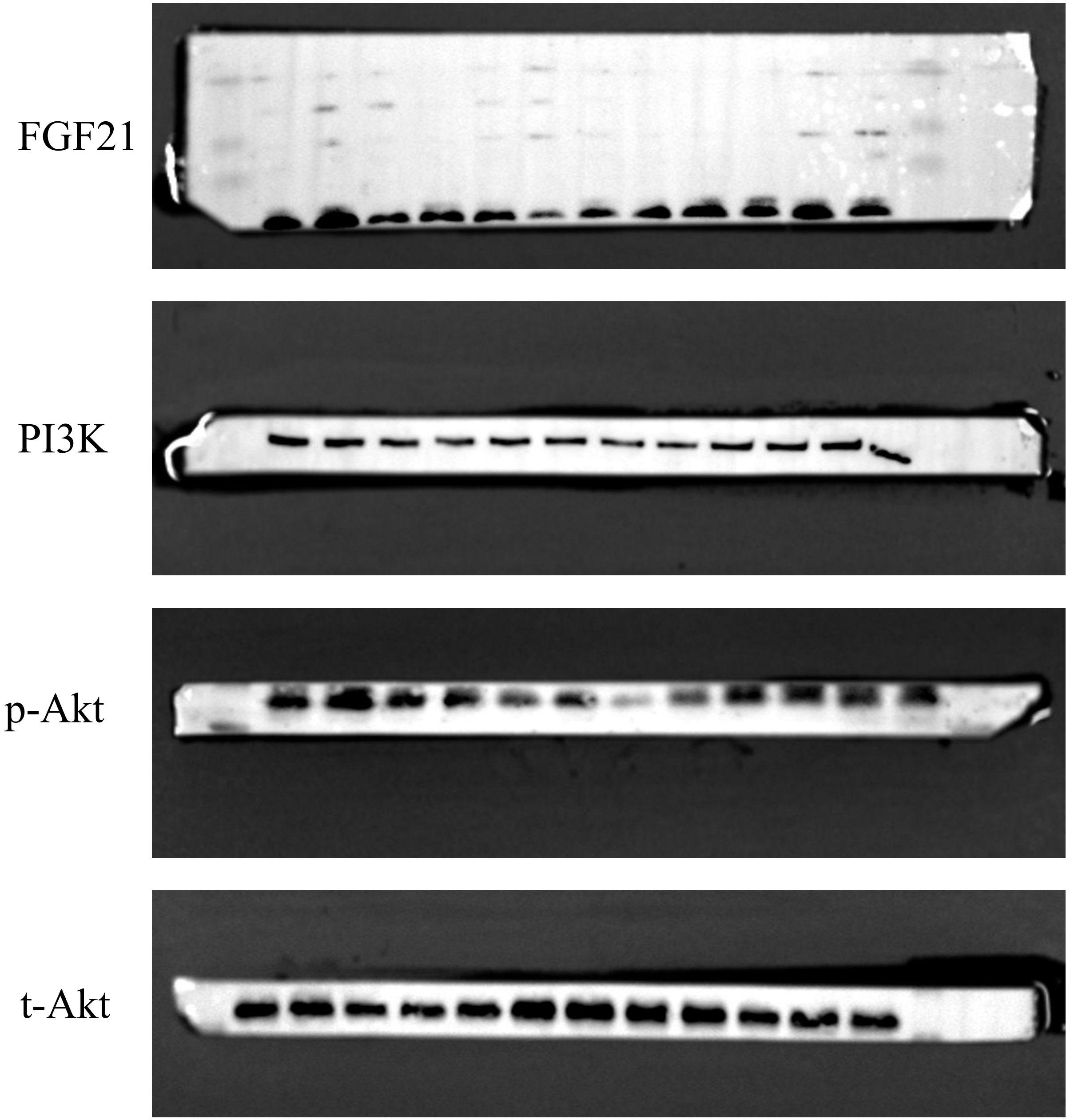

Figure 4

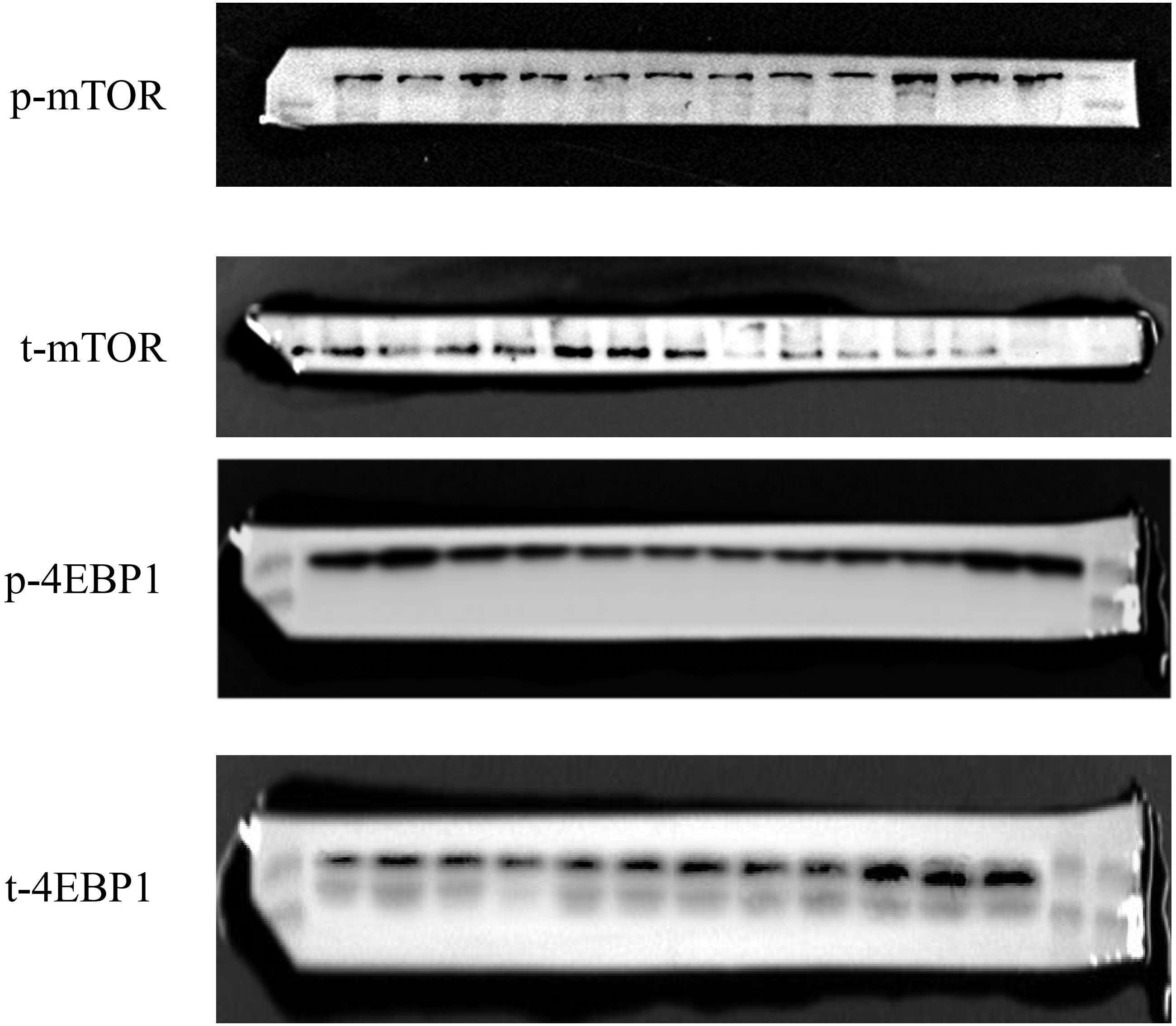

Figure 4

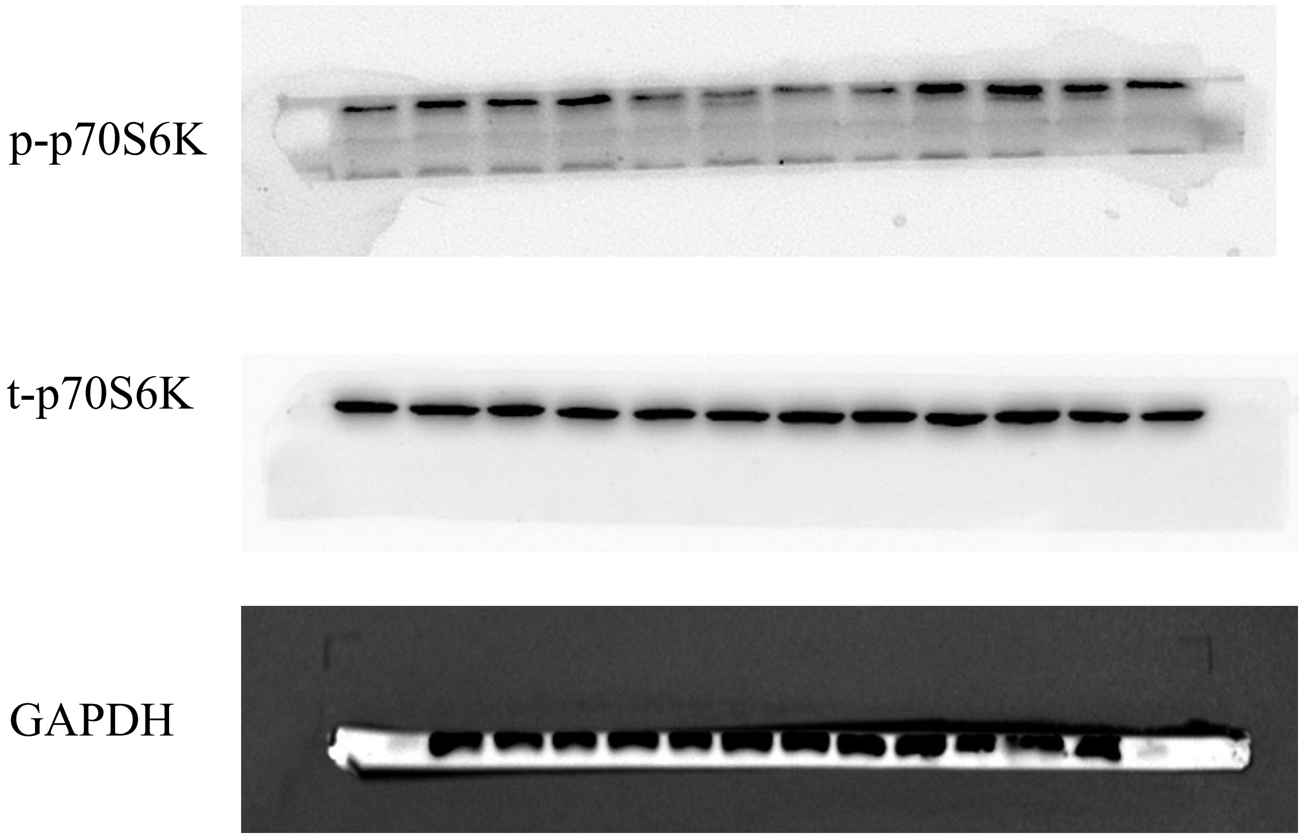

Figure 5

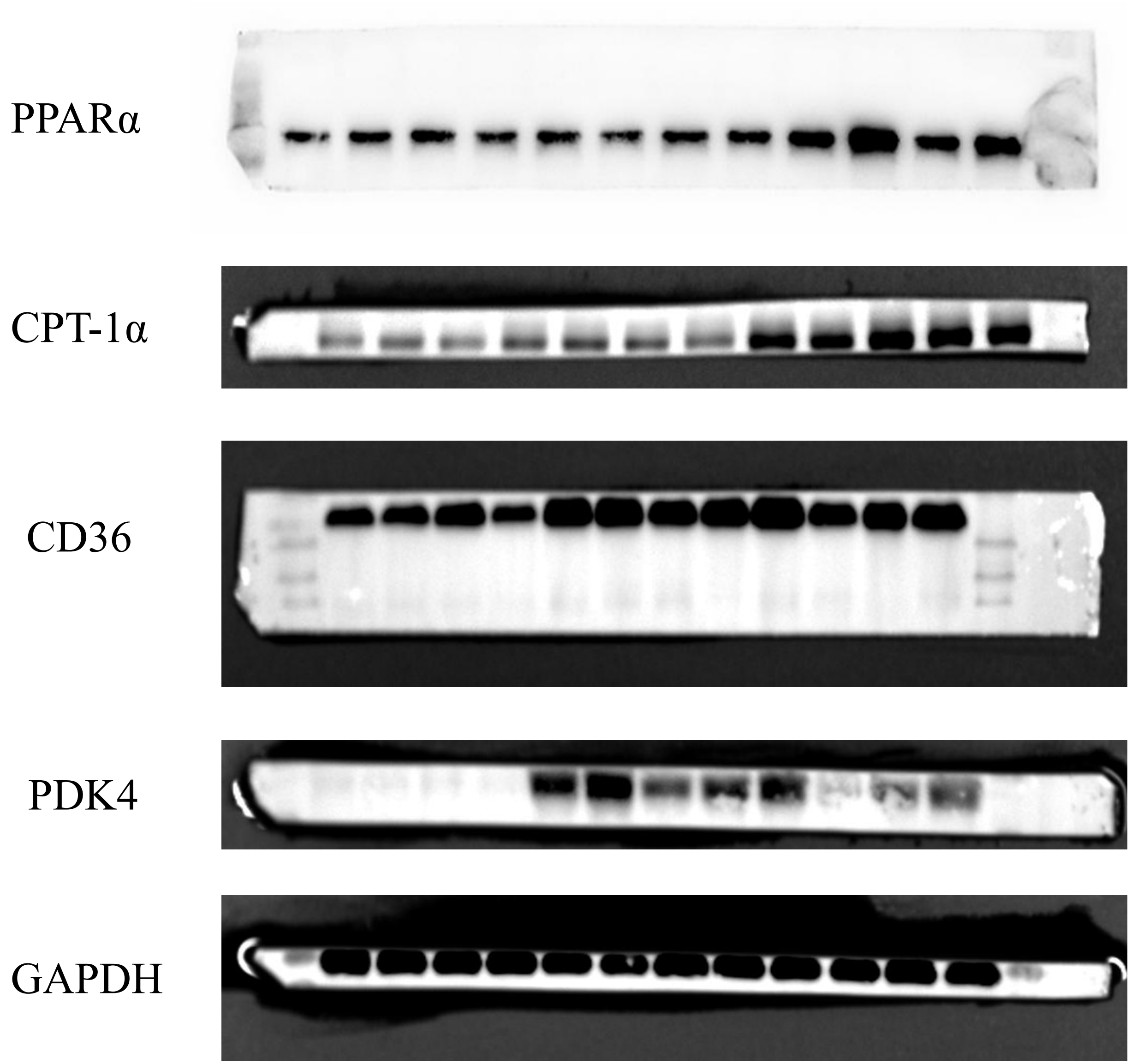

Figure 6

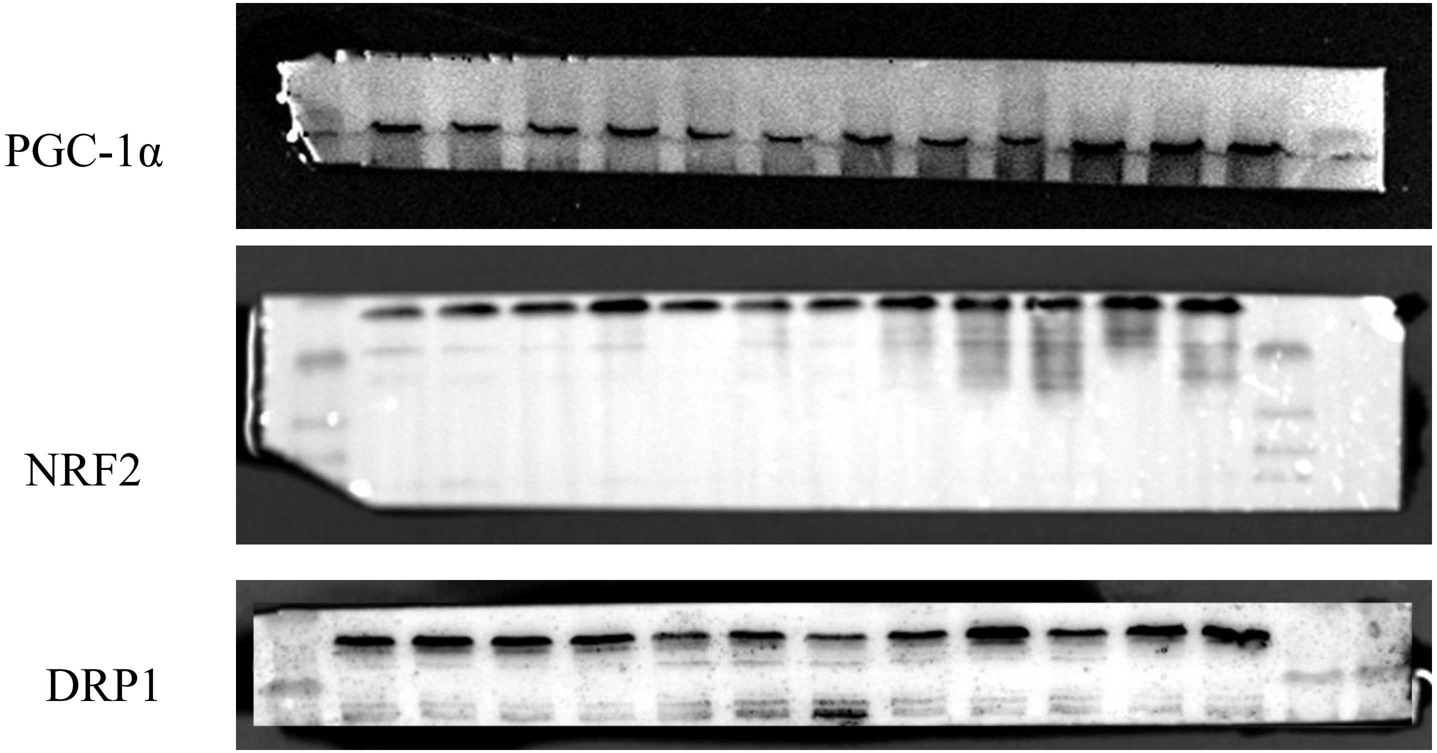

Figure 6

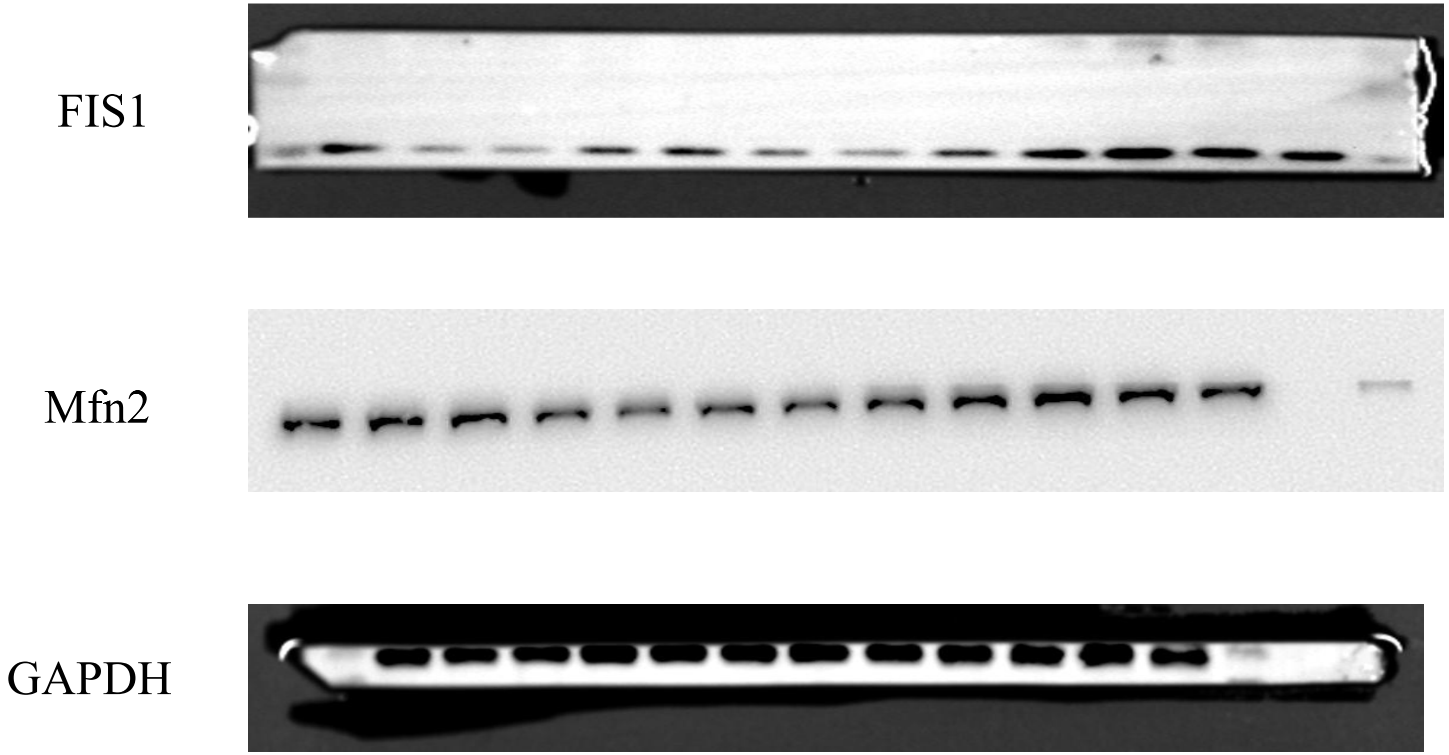

Supplement: Supplementary file 1 [file biomolecules-16-00003-s001.zip › Supplementary Materials File S1.pdf]
